# Generic drug prescribing in central Saudi Arabia: Perceptions and attitudes of physicians

**DOI:** 10.4103/0256-4947.51819

**Published:** 2009

**Authors:** Abdullah A. Alghasham

**Affiliations:** From the Department of Pharmacology and Therapeutics, College of Medicine, Qassim University, Buraidah, Saudi Arabia

## Abstract

**BACKGROUND AND OBJECTIVES:**

Physicians play a key role in generic drug prescribing, but their behavior is affected by many determinants. We examined physician practices and attitudes and other factors influencing the prescription of generic drugs.

**METHODS:**

A self-administered questionnaire was used to collect information from a random sample of physicians from different settings in central Saudi Arabia. Data were analyzed to describe all variables and test any significant difference between groups of physicians.

**RESULTS:**

The study included 772 physicians from different affiliations. The majority of physicians (n=741, 96%) reported that they knew enough about the therapeutic value of generic drugs. The majority (75%) reported that they knew the price differences, and this knowledge helped 72% of them to switch to generic prescription medication. Most physicians (79%) support generic substitution, but they indicated that there are certain clinical situations where they prefer to use brand name drugs. Physicians reported receiving visits and samples more frequently from representatives of brand name companies. Physicians did not report a significant difference in pressure from patients to prescribe either generic or brand drugs. Most physicians had a positive attitude towards the government role in assuring the quality of local drug products (80%) and in enforcing physicians to prescribe generic drugs (85%).

**CONCLUSION:**

Physicians face competing forces to prescribe either brand name or generic drugs. The majority support generic drug substitution. There are multiple factors that support prescription of generic drugs.

Health care costs have been on the rise globally, and this trend is expected to continue.^1^ Expenditures on pharmaceuticals are considered a major driving factor for rising health care costs. Spending on drug prescriptions has increased sharply in recent years with a growth rate faster than other major components of the health care system.^2^ The drug market in Saudi Arabia in 2006 exceeded 6 billion Riyals (1.6 billion US dollars), which accounts for 10% of health care expenditures.^3^ The growth rate of the drug market in Saudi Arabia exceeded 10% in the year 2006.^3^ Many countries have adopted cost-control strategies to slow drug spending growth such as drug utilization review and encouraging the use of less expensive generic drugs.^4^–^6^

The use of generic drugs can provide substantial savings in health care cost without affecting the quality or the therapeutic effect of the prescribed medicine. A generic drug is identical, or bioequivalent, to a brand name drug in dosage form, safety, strength, route of administration, quality, performance characteristics and intended use.^7^

The adoption of generic drugs in medical practices is a complex phenomenon and many determinants can affect it. Physicians play a key role in controlling this phenomenon and their decision in prescribing generic drugs is likely to be affected by many factors. Although there is increasing local and international encouragement for physicians to prescribe generic products, some physicians are not in favor of prescribing generic drugs. Therefore, many studies have tried to find determinants of this practice.^8^–^11^

In Saudi Arabia, generic drug prescribing has become a common practice; however, this trend has not been assessed. The purpose of this study is multifaceted. The study aimed to investigate physician perception and attitudes regarding substituting generic medications for brand name drugs and to examine factors that affect this pattern of prescription. Lastly, the study explored various types of potential pressures from patients and drug companies that might affect physician decision-making to prescribe generics.

## METHODS

Preliminary interviews were conducted with a sample of 12 physicians and 4 pharmacists to assess the significant dimensions of generic drug practices. Based on this initial response and a review of the literature, a questionnaire was designed and constructed. The questionnaire was refined after pre-testing it on a sample of physicians.

This study was conducted in central Saudi Arabia. All selected individuals were practicing physicians. Sampled physicians included primary health care, hospital, as well as private practitioners. The sample size was estimated to assess the perceptions and attitudes of physicians towards generic drug prescribing, with a prevalence rate of 15% and an absolute precision of 3% on both sides of this anticipated prevalence and a confidence level of 95%. The required sample size was 544 physicians. The sample was multiplied by 1.5 to accommodate for the design effect since sample selection was based on a stratified sampling method. The required sample after considering the design effect was 816 physicians. The sample size was increased to 900 to compensate for non-response. To obtain this sample, physicians were selected from primary health care centers, governmental hospitals and private sectors.

The questionnaire was designed to elicit the perception, attitude and behavior in relation to generic prescribing. To standardize the definition of generic drugs among study participants, a clear definition was included at the beginning of the questionnaire. The questionnaire collected information on: (1) personal, background and demographic data of the sampled physicians including their affiliation, specialty, years of experience and age; (2) the perception of physicians of price differences between generics and brand names and the sources of their information; (3) physician attitudes and beliefs regarding generic drug quality, therapeutic efficacy, the impact of use of generics on drug research, and the perception that the use of drugs has potential cost savings; (4) prescribing beliefs of physicians; and (5) the role of patients, drug company representatives, and health care administration policy in influencing the physician decisions on prescribing.

The questionnaire was self-administered and distributed by ordinary and electronic mail and by personal contact. The sample was a stratified sample to ensure representation of physicians from different settings and affiliations. Analysis of data was done using the Statistical Package for Social Sciences (SPSS 11.0). Frequency distributions of all variables were produced. All *P* values were based on 2-sided tests, and the cutoff value for statistical significance was set at.05. Chi-square analysis was used to test differences in proportions and an independent *t* test was used for analysis of quantitative data. For testing differences between proportions, the sample size was calculated to estimate a difference of 0.03 between proportions with a 5% level of significance and a power of 90% in a two-sided test. The estimated sample size was equal to more than 668 so the larger sample size (900) was used.

## RESULTS

Data were collected between May and September 2007. The response rate to the questionnaire was 85.8% (772 of 900) (Table 1). Primary care physicians were more likely to see more than 100 patients in a typical week compared with hospital and private physicians (92%, *45%, 25%,* respectively, *P*<.05). Furthermore, they were more likely to report seeing patients of all ages than hospital and private physicians (67%, 23%, 10%, respectively, *P*<.05). The number of prescriptions written by primary care physicians per week was also higher than that written by hospital and private physicians (median of 146, 74 and 92, respectively, *P*<.05).

**Table 1:** Personal, demographic and background data on the physicians participating in the survey.

Characteristics	Number (%)
Affiliation of physicians	
Primary health care	371 (48)
Private sector	108 (14)
Governmental hospitals	293 (38)
Total	772 (100)
	
Mean age (SD), year	48.7 (7.9)
	
Sex	
Male	659 (85)
	
Mean duration of practice (SD), years	22.2 (8.1)
	
Type of practice	
Clinical	733 (95)
Others	39 (5)
	
Patient load	
>60/week	38 (5)
60-100/week	502 (65)
<100/week	232 (30)
	
Patient type usually seen	
All age groups	525 (68)
Only children	54 (7)
Only adults	178 (23)
Only geriatrics	15 (2)
	
Median number of prescriptions written/physician per week	
Primary health care	146
Private sector	74
Governmental hospitals	92

The majority of physicians (n=741, 96%) reported that they knew enough about the therapeutic value of generic drugs they prescribe. There was no difference between primary care, hospital, and private physicians in their perception of knowledge about the therapeutic value of generic drugs (98.5%, 94.2% and 95%, respectively *P*>.05). Younger physicians (75% of those 30-40 years of age) were less likely than older physicians (93% of those 40-50 years and 89% of those ≥50 years of age) to report that they knew enough about the therapeutic value of generics to prescribe them and to illustrate this value to patients and peers (*P*<.05).

**Figure 1:**
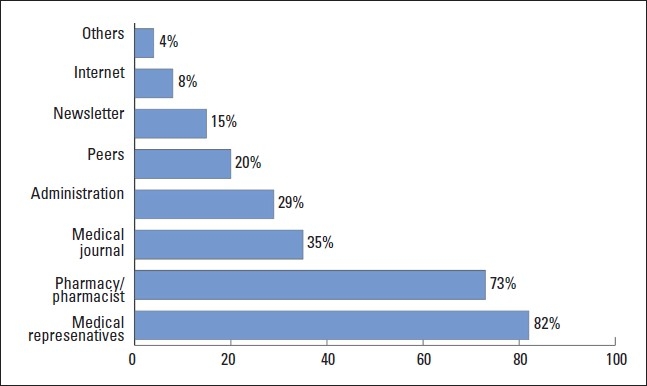
Source of information about generic alternatives to brand-name drugs.

The majority of physicians (75%) reported that they knew “some” about the price differences between brand name and generic drugs. On the other hand, 13% indicated that they knew “a lot” about the price differences. The rest of physicians either knew “very little” (7%) or knew “nothing at all” (5%). Also, the majority of physicians (72%) strongly agreed that the price difference helped them to switch to a generic prescription. Based on physician knowledge about the price difference, primary care physicians were significantly more likely to switch to generic prescription than hospital and private physicians (47%, 31% and 22%, respectively, *P*<.05). The main source of information about generic drugs were drug manufacturer representatives followed by pharmacists and medical journals (Figure 1). The drug formulary was the least source of information.

**Figure 2:**
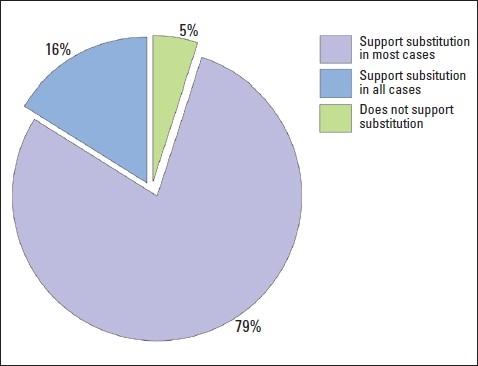
Proportions of physicians who support generic drug substitution.

The majority of physicians (79%) supported generic substitution in “most” cases, and reported that there are certain situations where brand drugs are preferred (Figure 2). Only 16% of the physicians supported using generics in “all” clinical situations if generic drugs were available. On the other hand, only 5% did not support generic substitution at all. The preference of generic substitution in “most” cases was more common among primary care physicians than hospital and private physicians (55%, 28%, 17%, respectively, *P*<.05). On the other hand, hospital physicians were more likely than primary care and private physicians (37%, 33%, 30%, respectively, *P*>.05) to support generic substitution in “all” cases where a generic drug is available. However these differences were not significant.

**Figure 3:**
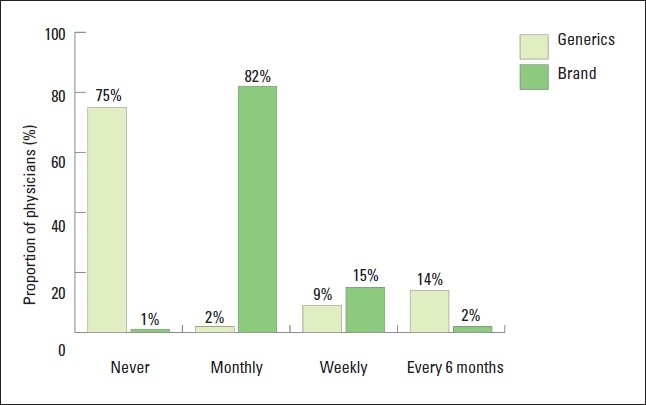
Proportions of physicians who have visits from generic and brand name companies' representatives.

Most physicians (82%) reported visits by representatives of brand name drug companies on a monthly basis (Figure 3). On the other hand, representatives of generic drug companies were less likely to visit physicians. Seventy-five percent of physicians claimed that they had never been visited by representative of generic drug companies. Ninety-seven percent of physicians who had been visited by brand name drug company representatives reported receiving samples while only 22% of received samples from generic drug companies (*P*<.05). There was no significant difference between primary care, hospital and private physicians in receiving visits and samples from representatives of generic drugs. However, private physicians were more likely than hospital and primary care physicians to have visits and samples from brand name company representatives.

**Figure 4:**
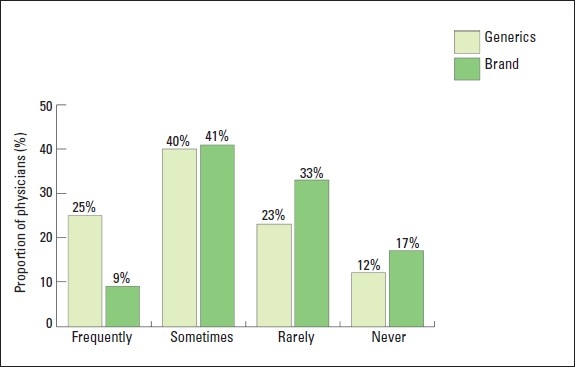
Proportions of physicians who feel pressured by patients to prescribe generic or brand drugs.

An equal proportion of physicians “sometimes” felt pressured by patients to prescribe either brand drugs or generic drugs (Figure 4) (41% vs. 40%, respectively, *P*>.05). However, physicians were more likely to report that they “frequently” felt pressured to prescribe generics (25%) rather than brand name drugs (9%, *P*<.05). Physicians reported that 23% of patients asked about the cost of the prescribed drug, 16% asked about effectiveness, and only 8% talked about their past experience with generic drugs. Overall, the proportion of physicians who expected the pharmacist to dispense brand name as written without substitution was 47%. However, hospital physicians (23%) were significantly less likely than primary care (35%) and private physicians (54%) to report that pharmacists would dispense brand drugs without substitutions (*P*<.05). Only 25% of physicians in governmental institutions (and centers) reported that the prescription form they use included a statement that allowed the pharmacist to substitute generic drugs when brand drugs were not available. None of the private physicians reported that the prescription forms they use included such a statement. Seventy-four percent of physicians expressed acceptance that the pharmacist could substitute a prescribed brand drug by a generic one while 21% accepted that the pharmacist could substitute a brand drug for a generic drug.

Most physicians had a positive attitude towards the government role in controlling the drug industry (82%), assuring the quality of drug products (80%), and enforcing physicians to prescribe generic drugs (85%). Most physicians (65%) believed that generic drugs produce the intended therapeutic effects, while 67% reported that the efficacy of generic drugs was equal to that of brand drugs (Table 2).

**Table 2:** Proportion of physicians who strongly or somewhat agree with the items related to government drug regulations.

Item	%
• I feel secure in prescribing drugs approved by government	82
• Government is capable of assuring high quality of local drug products	80
• Requirements for approval of generics is less than that for brand names	63
• Health care administration is forcing physicians to prescribe generic drugs	85
• Therapeutic failure is a serious problem of some generic drugs	35
• Generic drugs generally produces the indented therapeutic effects	65
• Most generic and brand names are equally effective	67

Seventy-two percent of physicians believed that price differences between brand and generic drugs helped them to switch to generics. The majority of physicians (84%) believed that using generics helps in saving health costs. A proportion of private physicians (24%) used generic drugs to help them keep their patients. Additionally, 61% of physicians believed that wider use of generic drugs would not affect the research and development of new drugs.

## DISCUSSION

The response rate of physicians to the questionnaire (85.8%) was satisfactory and comparable to other studies,^8^–^10^ but the characteristics of nonrespondents are unknown. Most physicians in this study were in their late forties in age. They were mainly practicing clinicians for an average period of experience that exceeded 20 years and saw more than 60 patients every week in all age groups. Characteristics of physicians regarding their demographic, personal and practice information were comparable with the data at the national level.^12^

Primary care physicians were more likely than hospital and private physicians to see more patients in a typical week, see patients of all ages, and write more prescriptions in a week. This observation is in parallel with the job requirements and policy planning for primary health care in Saudi Arabia; thus, primary care physicians constitute the majority of practicing physicians in Saudi Arabia.^12^,^13^ Therefore, their contribution in cost savings would be substantial if they switch to generic prescriptions.

The majority of physicians supported generic substitution in “most” cases. Other studies showed the same findings and supported generic substitution.^11^,^14^–^16^ In this study, “certain situations” where they prefered to use brand name drugs were not identified and it is difficult to know in which situations physicians will not support generic interchange. Interestingly, few physicians do not support substitution to generics at all. Reasons against supporting generic substitutions were not addressed in the study. This could be due to influence from the brand drug company's “war against generics”, lack of awareness about price differences, the belief that “genuine is better”, or due to previous experience with generics. Although switching to generic drugs is widely practiced worldwide, there are still certain situations where interchangeability is controversial. When switching from brand to generic drugs with a narrow therapeutic index or critical dosing, bioequivalence may not be guaranteed and interchange is not advisable.^17^

The majority of physicians also reported that they knew about the price difference between brand name and generic drugs. Also, they strongly agreed that the price difference helped them to switch to a generic prescription; this is similar to findings in other studies.^10^,^11^ It seems that whenever the knowledge is provided about generic drug prices, the required behavior can be ensured.^18^–^19^ Proper education and training programs for medical students and for practicing clinicians are recommended to raise the level of physician knowledge of the benefits of generics.

Although physicians reported that representatives of generic drugs do not visit them frequently, they selected them as the first source of their information about generics. Pharmacists were the second major source of information about generic drugs for physicians. Other valid and reliable sources such as medical journals, newsletters, the internet, and administration played a minor role. Therefore, the pharmacist contribution in providing valid information to physicians should be improved through training programs, workshops, and other active methods of education and learning.^20^ Physicians should take their information about drugs from reliable sources. Probably the best source of information about marketed drugs in Saudi Arabia is the Saudi National Formulary (SNF). It is available on-line and provides information and pricing on registered and marketed drugs in the country.

Physicians reported that they felt pressured by patients and the health care administration to prescribe generic drugs. Physicians also reported that patients wanted to talk about the side effects, the cost and the effectiveness of the prescribed drugs and their past experience with generic drugs. It seems that generic drug prescription in the clinical practice is the result of mutual and active interaction between the physician and the patient. Therefore, both physicians and patients can be empowered by reliable information so that they both can place adequate pressure and influence on the other party to influence generic prescription.^21^

Most physicians agreed that the pharmacist can substitute a generic for a brand drug, but do not agree with a brand drug in place of a generic one. Also, they expected the pharmacist to switch to a generic drug instead of the written brand name without permission. A statement on the prescription form that gives the pharmacist the right to substitute generic instead of brand name drugs is not common and is more likely in the hospital setting. All these conflicting observations underscore the vital role of the pharmacist in generic drug dispensing.^22^–^24^ Therefore, facilitating regulations, training programs and continuous monitoring of pharmacists should be an integral part of any cost saving program that encourages generic drug prescription. Efforts to educate physicians early in their career about the benefits and the value of generic drug prescribing should be encouraged. A viable role for other health care professionals should also be sought. Policy makers should continue to encourage generic prescribing when generic equivalent is available and suitable. Quality assurance of drugs, both brand and generics, should continue after approval through post-marketing surveillance.

The findings of this study indicate that physicians are faced by multiple and sometimes competing forces to prescribe either brand name or generic drugs. In this study, the majority of physicians support generic drug substitution. Supporting forces for generic prescription include physician knowledge about generic drug effectiveness and price differences, a positive attitude among physicians towards generic drugs, the influence of patients on prescribing generic drugs and the government role in supporting generic prescription. On the other hand, factors that work against generic prescription include the influence of the brand name drug companies and the use of drugs with a narrow therapeutic index. Further studies are needed to explore situations and factors where switching from brand to generic drugs may not be advised.
